# Intake of Coffee Associated With Decreased Depressive Symptoms Among Elderly Japanese Women: A Multi-Center Cross-Sectional Study

**DOI:** 10.2188/jea.JE20190010

**Published:** 2020-08-05

**Authors:** Yasumi Kimura, Hitomi Suga, Satomi Kobayashi, Satoshi Sasaki, the Three-Generation Study of Women on Diets and Health Study Group

**Affiliations:** 1 Department of Nutritional Sciences, Faculty of Nutritional Sciences, Nakamura Gakuen University, Fukuoka, Japan; 2 Department of Social and Preventive Epidemiology, School of Public Health, The University of Tokyo, Tokyo, Japan

**Keywords:** coffee, green tea, caffeine, depressive symptoms, elderly women, Japan

## Abstract

**Background:**

Depression in elderly people is a major global concern around the world. Epidemiological evidence of the association of beverages with depressive symptoms has received research attention; however, epidemiological studies on the association of coffee and green tea consumption with depressive symptoms among the elderly population are limited. The objective of this study is to cross-sectionally examine the association of depressive symptoms with the intake of coffee, green tea, and caffeine and to verify the antidepressant effect of caffeine.

**Methods:**

The subjects were 1,992 women aged 65–94 years. Intakes of coffee, green tea, and caffeine, as well as depressive symptoms, were assessed with a validated brief dietary history questionnaire (BDHQ) and the Center for Epidemiologic Studies Depression Scale (CES-D), respectively. Multiple logistic regression analysis was used to calculate odds ratios (ORs) and 95% confidence intervals (CIs) for depressive symptoms with adjustments for potential confounders.

**Results:**

Coffee intake was associated with a lower prevalence of depressive symptoms, the ORs of which for the 4th versus the 1st quartiles of intake was 0.64 (95% CI, 0.46–0.88, *P* for trend = 0.01) in a fully adjusted model. Caffeine intake was marginally associated with depressive symptoms, but the association was not statistically significant (OR 0.75; 95% CI, 0.55–1.02, *P* for trend = 0.058).

**Conclusion:**

The result suggests that the inverse association of coffee intake with depressive symptoms might be associated with not only caffeine intake but also some other substances in coffee or factors related to coffee intake. Because of the cross-sectional design of the present study, longitudinal studies are required to confirm the present finding.

## INTRODUCTION

Depression is an important public health concern, and late-life depression is associated with increased mortality.^1^ Lifetime prevalence of depressive symptoms is 14.6% in developed countries.^2^ In particular, women have a 2-fold increased risk of depression compared with men.^3^

Epidemiological evidence of the association of beverages with depressive symptoms has received research attention; however, epidemiological studies on the association of coffee and green tea with depressive symptoms in the elderly population are limited. Only one study has reported relations between coffee consumption and mental health^4^ in elderly community-dwelling subjects, and a relation concerning depressive symptoms does not appear to have been investigated. In the research for the non-elderly population, evidence has accumulated indicating that consuming high amounts of coffee is associated with lower depressive symptoms^5^^–^^14^; however, no association^15^^,^^16^ and a positive association^17^ have also been indicated. Elderly subjects might have different tendencies than non-elderly population. Thus, the relation between coffee consumption and depressive symptoms in community-dwelling elderly women, in whom this condition is highly prevalent, remains unclear. Several studies have shown that higher amounts of green tea are related to a lower prevalence of depressive symptoms^4^^,^^8^^,^^10^ or psychological distress,^18^ whereas green tea was not associated with mental health.^19^ Previous studies have reported significant inverse associations between green tea intake and depressive symptoms in a Japanese working population^8^ and among Japanese community-dwelling elderly subjects.^4^

Coffee and tea are two of the most widely consumed beverages in the world.^20^^,^^21^ In Japan, 47% and 53% of adults drink coffee and green tea, respectively, every day.^22^ Both coffee and green tea contain many biological active constituents, including polyphenols and alkaloids^23^^,^^24^; caffeine in coffee or green tea is widely used as a central nervous system stimulant.^25^ It is expected that coffee, green tea, and caffeine intake could be effective for the prevention of depressive symptoms. The relationship between caffeine intake and depressive symptoms has been examined in Western countries, and an inverse association has been reported.^6^^,^^10^^,^^26^ However, studies in Japan^8^^,^^19^ showed no association between caffeine and depression^8^ or mental health.^19^ These inconsistent results may be because the sources of caffeine among Japanese people are different from those in Westerners.^27^ In the United States, the main sources of caffeine are coffee (71%), soft drinks (16%), and tea (12%).^28^ However, in Japan, the primary sources of caffeine are coffee (46.7%), soft drinks (0.8%), and Japanese and Chinese tea (47.1%).^27^ Accordingly, it is possible that compounds in coffee other than caffeine are responsible for suppressing depression.^28^

By examining the relationship between coffee, green tea, or caffeine intake and depression, we sought to clarify whether the component with the depression suppressive effect is caffeine. In the present study, we aimed to investigate the association of coffee, green tea, and caffeine on depressive symptoms in a large-scale study among elderly women.

## METHODS

### Study procedure and subjects

The study was based on a cross-sectional multicenter survey, of which details have already been published.^29^ Briefly, participants of the survey were first-year students of dietetic courses at universities, colleges, and technical schools, along with their mothers and grandmothers. The survey was conducted from April to May in 2011 and 2012 in Japan. Since the Great East Japan Earthquake occurred in March 2011, surveys in the northeastern part of Japan have been difficult to conduct, and so the survey was conducted from April to May 2011 in Hokkaido and the southwestern part of Japan. Then, the same survey was conducted from April to May 2012 in the northeastern part of Japan. The research is not a clinical trial and does not need to be registered.

**Figure 1.**
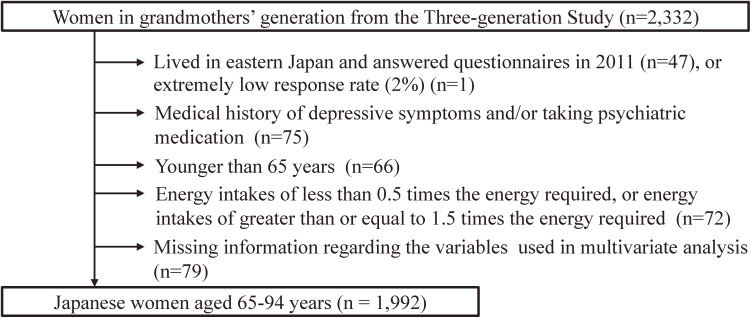
Exclusion criteria for association between intake of coffee and depressive symptoms among elderly Japanese women

The overall purpose of the survey was to examine the association between lifestyle, including diet, and health problems. In total, 85 teaching institutions participated and 7,016 first-year students were provided with questionnaires. Students were required to distribute questionnaires directly to their mothers and grandmothers, and those who were unable to do so were excluded from participation, except in the case that grandmothers were unavailable (65–89-year-old female acquaintances were allowed instead of grandmothers). The subjects of this study were the grandmothers’ generation (*n* = 2,332). We excluded subjects who lived in eastern Japan and who completed the questionnaire in 2011 (*n* = 47), because we assumed that they could not detail their usual dietary habits and lifestyle owing to the occurrence of The Great East Japan Earthquake in March 2011. We also excluded a subject in an institution in which the response rate was extremely low (*n* = 1). We further excluded those with a medical history of depressive symptoms (*n* = 43) and those on psychiatric medication (*n* = 62). Further, we excluded subjects aged <65 years (*n* = 66) and those with a reported energy intake of less than half of the requirement for the lowest physical activity category according to the Dietary Reference Intakes for Japanese, 2015 (<825 kcal/day for age 65–69 years: *n* = 6, <750 kcal/day for age >70 years: *n* = 16) or more than 1.5 times the energy requirement for the highest physical activity category (<3,300 kcal/day for age 65–69 years: *n* = 2, <3,000 kcal/day for age >70 years: *n* = 48),^30^ as well as those with missing information on the variables of multivariate analysis (*n* = 79). Some participants met two or more exclusion criteria. After these exclusions, 1,992 women aged 65–94 years remained (Figure 1). Surveys at participating institutions were conducted according to the survey protocol, which was approved by the ethics committee of the Faculty of Medicine, The University of Tokyo (No. 3249).

### Ascertainment of depressive symptoms

Depressive symptoms were assessed using a Japanese version^31^ of the Center for Epidemiologic Studies Depression (CES-D) scale.^32^ The detail of the questions of CES-D is written in the previous report.^31^ In brief, the CES-D scale includes 20 questions marked between 0 and 3, with higher scores indicating that the situation or condition appears more frequently (score 0 = never and score 3 = always), except for questions 4, 8, 12, and 16, which were scored in reverse order (score 3 = never and score 0 = always); the total score can range from 0 to 60. The scale addressing depressive symptoms referenced subjects’ experiences during the preceding week. We used CES-D score of ≥16 as a cutoff value to define depressive symptoms. The criterion validity of the CES-D scale has been well established.^31^^,^^32^

### Dietary assessment

Dietary intake during the preceding 1-month period were assessed using a validated brief dietary history questionnaire (BDHQ),^33^ covering 58 food and beverage items. The BDHQ is a structured questionnaire that includes questions regarding the intake frequencies of selected foods commonly consumed in Japan (a sample copy of the BDHQ is available at http://www.nutrepi.m.u-tokyo.ac.jp/dhq/BDHQ1-1.pdf). Spearman correlation coefficients between intakes of coffee and green tea according to the above-mentioned BDHQ and those from 16-day dietary records were 0.77 and 0.64 for coffee and green tea intake, respectively, in women.^34^ Nutrient and caffeine intake from diet was estimated using an *ad hoc* computer algorithm with reference to the Standard Tables of Food Composition in Japan^35^ and caffeine composition database developed by Yamada et al.^27^ We calculated total caffeine intake estimated from BDHQ with foods, coffee, and green tea, since there are several sources of caffeine in foods other than coffee and green tea, such as confectionaries, black tea, Chinese tea, soft drinks, and cocoa.^27^ The Spearman correlation coefficient of the self-administered diet history questionnaire (longer version of the BDHQ) and 16-day diet records was 0.37 among 92 Japanese women for total caffeine intake, (S. Sasaki, unpublished observation, 2014). In the BDHQ, coffee and green tea are asked as independent food items. Therefore, accurate intake of coffee and green tea can be calculated separately. Nutrient and food intake were energy-adjusted using the density method.^36^ Alcohol intake (yes or no), eicosapentaenoic acid (EPA), docosahexaenoic acid (DHA), and folate intakes were assessed in the BDHQ. Although dietary supplement use was assessed in the lifestyle questionnaire, because of the lack of reliable information about the composition table of dietary supplements in Japan, the intake from supplement was not included in the nutritional value calculation.

### Other variables

Age, body height, and body weight were self-reported in the BDHQ. Body mass index (BMI) was calculated as body weight (kg) divided by the square of body height (m). The lifestyle questionnaire included questions about the residential block (Hokkaido and Tohoku, Kanto, Hokuriku and Tokai, Kinki, Chugoku and Shikoku, and Kyushu), and size of residential area (city with a population ≥1 million, city with a population <1 million, and town and village). Physical activity measured by metabolic equivalents per hour (METs/day) was calculated from the time spent on the five types of physical activity (walking, bicycle riding, running, standing, and playing sports), sitting, and sleeping. The duration of the five types of physical activities and sleeping was self-reported in the lifestyle questionnaire. Sitting duration was calculated by subtracting the sum of the duration of the five physical activities and sleeping from 24 hours. MET values for each activity were as follows: walking (3.5 METs/hour), bicycle riding (7.5 METs/hour), standing (3.2 METs/hour), running (7.0 METs/hour), playing sports (8.0 METs/hour), sleeping (1.0 METs/hour), and sitting (1.3 METs/hour).^37^ These METs were multiplied by the time spent for each activity, and the sum of these yielded the extent of physical activity, expressed as METs/day. The lifestyle questionnaire also included inquiries about marital status (single, married, widowed, and separated), living status (alone, not alone), current smoking status, educational level (≤ junior high school, high school, junior college, and ≥ university and higher), and dietary supplement intake.

### Statistical analysis

Study participants were divided into quartiles according to coffee and green tea intake, and the baseline characteristics were evaluated using linear regression analysis for continuous variables and the Mantel-Haenszel chi-square test of trend for categorical variables. Crude and multiple logistic regression analyses were used to calculate odds ratios (ORs) and 95% confidence intervals (CIs) of depressive symptoms for groups of coffee, green tea, and caffeine intake. The reference category was the lowest. We performed three types of analysis: 1) a crude model, 2) an age-adjusted model, 3) a multivariate model (model 1) adjusted for age (year, continuous), residential block (Hokkaido and Tohoku, Kanto, Hokuriku and Tokai, Kinki, Chugoku and Shikoku, and Kyushu), living status (alone or not alone), current smoking (yes or no), alcohol drinking (yes or no), marital status (married or unmarried), physical activity level (total metabolic equivalents tasks [METs]/day, continuous), size of residential area (city with a population ≥1 million, city with a population <1 million, and town and village), BMI (kg/m^2^, continuous), and education (junior high school, high school junior college, and university and higher); 4) a multivariate model (model 2) adjusted for the factors in model 1 plus EPA+DHA intake (mg/1,000 kcal, continuous), folate intake (µg/1,000 kcal, continuous), dietary supplement use (yes or no); and 5) a multivariate model (model 3) adjusted for the factors in model 2 plus mutually adjusted for green tea intake (g/1,000 kcal) or coffee intake (g/1,000 kcal). We included these variables in the model based on their known or potential relations to depressive symptoms. Regarding EPA+DHA, folate was adjusted because it related to depression in our previous study.^38^ Statistical significance was declared when *P* was less than 0.05. All statistical analyses were performed with SAS 9.1 (SAS Institute, Cary, NC, USA).

## RESULTS

The prevalence of depressive symptoms was 22.0% in this study. Characteristics of study participants according to green tea and coffee intake are shown in Table 1. Participants with higher intake of green tea had higher mean age and lower mean BMI. Those with higher green tea intake tended to consume higher amounts of caffeine and folate. Participants who consumed more coffee were younger and more likely to be physically active, current smokers, and current alcohol drinkers, and more likely to use dietary supplement. Those with higher coffee intake tended to show a higher intake of caffeine and lower intake of EPA+DHA and folate.

**Table 1.** Characteristics of study participants according to green tea and coffee intake: elderly Japanese women (*n* = 1,992)

	Green tea intake (*n* = 1,992)					Coffee intake (*n* = 1,992)				
		
	Quartile 1	Quartile 2	Quartile 3	Quartile 4	Trend *P*^a^	Quartile 1	Quartile 2	Quartile 3	Quartile 4	Trend *P*^a^
	(*n* = 498)	(*n* = 498)	(*n* = 498)	(*n* = 498)		(*n* = 498)	(*n* = 498)	(*n* = 498)	(*n* = 498)	
CES-D score	11.2 (6.7)^b^	11.2 (6.6)	11.0 (6.6)	10.4 (6.8)	0.047	11.9 (7.5)	10.7 (6.2)	11.1 (6.3)	10.2 (6.5)	0.002
Median intake, g/1,000 kcal	22 (0–99)^c^	190 (100–231)	273 (232–319)	390 (320–788)		0 (0–3)	21 (4–58)	81 (59–106)	194 (107–619)	
Age, years	73.9 (4.8)	74.6 (5.0)	74.9 (4.7)	75.2 (5.3)	<0.0001	76.4 (5.3)	74.8 (4.8)	74.3 (4.6)	73.0 (4.5)	<0.0001
BMI, kg/m^2^	23.1 (3.3)	22.7 (3.1)	22.6 (2.9)	22.6 (3.2)	0.02	22.4 (3.1)	23.0 (3.2)	22.8 (3.1)	22.6 (3.1)	0.45
Residential block, %										
Hokkaido and Tohoku	13.7	8.0	6.4	8.2	0.26	10.4	8.4	10.6	6.8	0.006
Kanto	11.8	27.7	29.3	31.7		30.5	31.3	22.9	15.9	
Hokuriku and Tokai	26.9	25.9	21.3	21.5		21.9	22.5	24.1	27.1	
Kinki	15.3	10.4	11.7	13.3		7.6	11.5	14.7	16.9	
Chugoku and Shikoku	24.5	16.3	13.6	9.2		10.2	10.0	18.1	25.3	
Kyushu	7.8	11.7	17.7	16.1		19.3	16.3	9.6	8.0	
Size of residential area, %										
City with a population ≥1 million	13.2	14.3	14.5	10.1	0.93	9.8	14.3	15.1	12.9	0.22
City with a population <1 million	75.1	73.1	75.5	80.3		78.7	75.1	73.5	76.7	
Town and village	11.7	12.6	10.0	9.6		11.5	11.6	11.5	10.4	
Married, %	60.0	64.1	60.6	58.6	0.44	60.4	58.0	61.9	63.1	0.23
Living status (alone), %	14.6	13.5	18.5	18.7	0.02	18.7	14.3	17.3	15.1	0.29
Physical activity, METs/day	39.0 (6.4)	39.3 (6.6)	39.2 (6.1)	38.5 (6.6)	0.18	37.8 (6.5)	39.1 (6.3)	39.1 (6.2)	40.0 (6.4)	<0.0001
Current smoking, %	3.2	2.6	1.6	2.4	0.27	1.8	2.0	2.2	3.8	0.04
Current alcohol intake, %	20.3	21.3	17.3	18.7	0.26	11.2	18.9	23.3	24.1	<0.0001
Education, %										
Junior high school	50.2	45.6	39.8	48.6	0.81	48.8	49.4	44.6	41.4	0.004
High school	38.6	46.2	48.8	45.2		44.4	41.0	45.2	48.2	
Junior college	9.8	7.6	9.2	6.0		5.6	9.0	8.8	9.2	
University or higher	1.4	0.6	2.2	00.2		1.2	0.6	1.4	1.2	
Caffeine intake, mg/1,000 kcal^d^	96.4 (74.3)	159.1 (59.7)	198.2 (58.0)	272.2 (79.8)	<0.0001	141.3 (80.5)	153.0 (76.6)	170.9 (69.1)	260.8 (95.5)	<0.0001
EPA+DHA intake, mg/1,000 kcal	609.2 (338.7)	679.5 (382.0)	656.8 (331.5)	605.8 (330.0)	0.64	672.0 (393.4)	655.3 (325.5)	644.0 (324.2)	579.9 (303.8)	<0.0001
Folate intake, µg/1,000 kcal	200.2 (68.4)	227.2 (61.7)	256.8 (68.7)	273.7 (72.8)	<0.0001	245.1 (82.6)	243.2 (69.8)	237.1 (65.8)	232.4 (71.9)	0.003
Dietary supplement use, %	28.3	32.7	30.1	29.3	0.97	25.9	30.1	30.3	34.1	0.007

BMI, body mass index; CES-D, Center for Epidemiologic Studies Depression Scale; DHA, docosahexaenoic acid; EPA, eicosapentaenoic acid; METs, metabolic equivalent hours.

Participants with depressive symptoms were defined as a CES-D score ≥16.

^a^Trend *P* values were based on linear regression analysis for continuous variables with ordinal numbers 0–3 assigned to green tea and coffee intake categories, or Mantel-Haenszel chi-square test for categorical variables.

^b^Values for continuous variables are in mean ± standard deviation (all such values).

^c^Range.

^d^Calculated from green tea, black tea and Chinese tea, coffee and cola.

The ORs and 95% CIs for depressive symptoms according to the quartile of intake of green tea, coffee intake are shown in Table 2. After adjustment for demographic, lifestyle, and socioeconomic factors (model 1), compared with subjects with the lowest quartile of coffee intake, those with the highest quartile had 34% significantly lower OR of depressive symptoms. Additional adjustment for dietary factors (model 2 and model 3) did not materially change the results. Green tea intake was not significantly associated with the depressive symptoms. The multivariate-adjusted ORs for depressive symptoms according to green tea intake of first, second, third, and fourth quartiles were 1.00 (reference), 1.28 (95% CI, 0.94–1.75), 1.06 (95% CI, 0.76–1.46) and 0.85 (95% CI, 0.62–1.17), respectively (model 3, *P* for trend = 0.16). In the analysis, the lowest quartile contains not only non-green tea drinkers or non-coffee drinkers but also some drinkers. Additionally, we analyzed using non-green tea drinkers (*n* = 156, 7.8%) or non-coffee drinkers (*n* = 491, 24.6%) as a reference, but the results did not change (eTable 1 and eTable 2). We further adjusted for cancer, diabetes, and ADL decline, which could be risk factors for depressive symptoms, and the multivariate-adjusted ORs for depressive symptoms according to green tea, coffee, and caffeine of the 4th vs the 1st quartiles of intake were 0.96 (95% CI, 0.70–1.33, *P* for trend = 0.56), 0.69 (95% CI, 0.50–0.96, *P* for trend = 0.048), and 0.85 (95% CI, 0.62–1.17, *P* for trend = 0.29), respectively. The results also did not change (data not shown in table). Concerning caffeine intake (Table 3), participants in the highest versus the lowest quartiles of caffeine intake had 25% lower odds of having depressive symptoms in a fully adjusted model (OR 0.75; 95% CI, 0.55–1.02); however, this association was not statistically significant. There were non-significant linear trend associations between intake of caffeine and likelihood of depressive symptoms (*P* for trend = 0.058). The correlation coefficients of coffee, green tea, and caffeine were *r* = 0.54 for green tea and caffeine, *r* = 0.52 for coffee and caffeine, and *r* = 0.12 for green tea and coffee.

**Table 2.** Adjusted odds ratio (95% CI) of depressive symptoms according to intake of green tea and coffee in elderly Japanese women (*n* = 1,992)

	Green tea intake (*n* = 1,992)					Coffee intake (*n* = 1,992)				
		
	Quartile 1	Quartile 2	Quartile 3	Quartile 4	Trend *P*^a^	Quartile 1	Quartile 2	Quartile 3	Quartile 4	Trend *P*^a^
	(*n* = 498)	(*n* = 498)	(*n* = 498)	(*n* = 498)		(*n* = 498)	(*n* = 498)	(*n* = 498)	(*n* = 498)	
Median intake, g/1,000 kcal	22 (0–99)	190 (100–231)	273 (232–319)	390 (320–788)		0 (0–3)	21 (4–58)	81 (59–106)	194 (107–619)	
Depressive symptoms, %	22.0	24.5	21.3	20.3		28.5	20.3	21.1	18.3	
Crude OR (95% CI)	1.00 (Reference)	1.14 (0.85–1.54)	0.95 (0.71–1.29)	0.90 (0.66–1.22)	0.30	1.00 (Reference)	0.63 (0.47–0.84)	0.66 (0.49–0.88)	0.56 (0.42–0.76)	0.0003
Age adjusted OR (95% CI)	1.00 (Reference)	1.11 (0.83–1.50)	0.91 (0.67–1.23)	0.84 (1.03–1.07)	0.15	1.00 (Reference)	0.68 (0.50–0.90)	0.71 (0.53–0.96)	0.64 (0.47–0.87)	0.008
Model 1^b^ OR (95% CI)	1.00 (Reference)	1.14 (0.84–1.54)	0.94 (0.69–1.28)	0.82 (0.60–1.12)	0.12	1.00 (Reference)	0.68 (0.50–0.92)	0.73 (0.54–0.99)	0.66 (0.48–0.91)	0.01
Model 2^c^ OR (95% CI)	1.00 (Reference)	1.28 (0.94–1.75)	1.07 (0.78–1.48)	0.87 (0.64–1.19)	0.21	1.00 (Reference)	0.70 (0.52–0.95)	0.75 (0.55–1.02)	0.65 (0.47–0.89)	0.01
Model 3^d^ OR (95% CI)	1.00 (Reference)	1.28 (0.94–1.75)	1.06 (0.76–1.46)	0.85 (0.62–1.17)	0.16	1.00 (Reference)	0.70 (0.52–0.95)	0.73 (0.54–1.00)	0.64 (0.46–0.88)	0.01

BMI, body mass index; CES-D, Center for Epidemiologic Studies Depression Scale; DHA, docosahexaenoic acid; EPA, eicosapentaenoic acid; METs, metabolic equivalent hours.

Participants with depressive symptoms were defined as a CES-D score ≥16.

Green tea, coffee intake, EPA + DHA intake and folate intake were energy-adjusted according to the density method.

^a^Trend *P* values were based on linear regression analysis for continuous variables with ordinal numbers 0–3 assigned to green tea or coffee intake categories.

^b^Model 1: adjusted for age (years, continuous) and residential block (Hokkaido and Tohoku, Kanto, Hokuriku and Tokai, Kinki, Chugoku and Shikoku, and Kyushu), living status (alone or not alone), current smoking (yes or no), alcohol drinking (yes or no), marital status (married or unmarried), physical activity level (total metabolic equivalents hours/day: METs, continuous), size of residential area (city with a population ≥1 million, city with a population <1 million, and town and village), BMI (kg/m^2^, continuous), and education (junior high school, high school junior college, and university and higher).

^c^Model 2: adjusted for variables in model 1 with EPA + DHA intake (mg/1,000 kcal, continuous), folate intake (µg/1,000 kcal, continuous), and dietary supplement use (yes or no).

^d^Model 3: adjusted for variables in model 2 with mutually adjusted for green tea intake (g/1,000 kcal) or coffee intake (g/1,000 kcal).

**Table 3.** Adjusted odds ratio (95% CI) of depressive symptoms according to caffeine intake in elderly Japanese women (*n* = 1,992)

	Caffeine intake (*n* = 1,992)				

	Quartile 1	Quartile 2	Quartile 3	Quartile 4	Trend *P*^a^
	(*n* = 498)	(*n* = 498)	(*n* = 498)	(*n* = 498)	
Median caffeine intake, mg/1,000 kcal	76.5 (0–119.2)	150.2 (119.3–173.0)	203.7 (173.3–234.8)	284.5 (234.9–758.0)	
Depressive symptoms, %	24.9	22.7	21.1	19.5	
Crude OR (95% CI)	1.00 (Reference)	0.89 (0.66–1.19)	0.81 (0.60–1.08)	0.73 (0.54–0.99)	0.03
Age adjusted OR (95% CI)	1.00 (Reference)	0.89 (0.67–1.20)	0.83 (0.62–1.12)	0.76 (0.56–1.03)	0.07
Model 1^b^ OR (95% CI)	1.00 (Reference)	0.91 (0.68–1.23)	0.86 (0.63–1.16)	0.74 (0.54–1.01)	0.052
Model 2^c^ OR (95% CI)	1.00 (Reference)	0.99 (0.73–1.33)	0.91 (0.67–1.24)	0.75 (0.55–1.02)	0.058

BMI, body mass index; CES-D, Center for Epidemiologic Studies Depression Scale; DHA, docosahexaenoic acid; EPA, eicosapentaenoic acid; METs, metabolic equivalent hours.

Participants with depressive symptoms were defined as a CES-D score ≥16.

Caffeine intake calculated from green tea, black tea and Chinese tea, coffee and cola.

Caffeine intake, EPA + DHA intake and folate intake were energy-adjusted according to the density method.

^a^Trend *P* values were based on linear regression analysis for continuous variables with ordinal numbers 0–3 assigned to caffeine intake categories.

^b^Model 1: adjusted for age (years, continuous) and residential block (Hokkaido and Tohoku, Kanto, Hokuriku and Tokai, Kinki, Chugoku and Shikoku, and Kyushu), living status (alone or not alone), current smoking (yes or no), alcohol drinking (yes or no), marital status (married or unmarried), physical activity level (total metabolic equivalents hours/day: METs, continuous), size of residential area (city with a population ≥1 million, city with a population <1 million, and town and village), BMI (kg/m^2^, continuous), and education (junior high school, high school junior college, and university and higher).

^c^Model 2: adjusted for variables in model 1 with EPA + DHA intake (mg/1,000 kcal, continuous), folate intake (µg/1,000 kcal, continuous), and dietary supplement use (yes or no).

## DISCUSSION

In this cross-sectional study among elderly Japanese women, we found an inverse association between coffee intake and depressive symptoms. A higher coffee intake showed 36% lower odds of having depressive symptoms in a fully adjusted model (OR 0.64; 95% CI, 0.46–0.88, *P* for trend = 0.01). Caffeine intake was marginally associated with depressive symptoms, but the association was not statistically significant (OR 0.75; 95% CI, 0.55–1.02, *P* for trend = 0.058). No significant association was found between green tea intake and depressive symptoms (OR 0.85; 95% CI, 0.62–1.17, *P* for trend = 0.16). This study is the first to report a significantly decreased prevalence of depressive symptoms among elderly Japanese women with a higher coffee intake.

The present study found a significantly lower prevalence of depressive symptoms among subjects with higher coffee intake in an elderly population. In previous research, only one study has reported relations between coffee consumption and mental health^4^ in elderly community-dwelling subjects, and no association with depressive symptoms has been observed. Our finding of an inverse association between coffee intake and depressive symptoms is closely in agreement with the results of the Nurses’ Health Study,^6^ in which those who consumed ≥4 cups/day coffee had a 20% lower prevalence of depressive symptoms compared with those consuming ≤1 cup/week. Similarly, studies in the United States showed 25% decreased depression among heavy (>813 mL/day) coffee drinkers^5^ and 10% decreased depression in those consuming ≥4 cups/day.^7^ In a Spanish cohort study, participants who drank ≥4 cups/day coffee showed a lower risk of depression (HR 0.37; 95% CI, 0.15–0.95).^11^ Likewise, a Japanese study showed a 39% lower odds of depressive symptoms who consumed ≥2 cups/day compared with those consuming <1 cup/day.^8^ Moreover, two Korean studies reported 42%^9^ and 32%^10^ lower odds of depressive symptoms. Furthermore, three systematic review and meta-analysis articles of observational studies reported a protective effect of coffee intake^12^^–^^14^; those with higher intakes had RR of depressive symptoms of 0.76 (95% CI, 0.64–0.91),^12^ 0.73 (95% CI, 0.59–0.90),^13^ and 0.76 (95% CI, 0.62–0.92).^14^ Conversely, a Canadian study in female participants who drank coffee in the amount of ≥4 cups/day and showed an increased risk of major depression had an OR of 1.38 (95% CI, 1.15–1.64) compared with non-coffee drinkers.^17^ Finnish^15^ and Japanese^4^ studies found no association between coffee intake and depressive symptoms; compared with not-daily drinkers, daily coffee drinkers had an OR of 0.90 (95% CI, 0.54–1.50) among adults aged 25–64 years^15^ and who consumed ≥1 cup/day had an OR of 0.82 (95% CI, 0.53–1.27), including elderly participants aged ≥70 years.^4^ The reason why the relation was not recognized might be that the exact coffee intake could not be evaluated because of the limitation on the choices of questionnaire about coffee intake (daily vs not daily,^14^ almost never, and ≥1 cup/day^4^). Regarding the positive association in the Canadian study,^17^ depressed subjects may be consuming more coffee as a form of self-medication.^39^ These inconsistent results might be explained by over- or underestimation of coffee intake due to differences in the dietary assessment methods and the possibility of misclassification of diagnosis criteria for depressive symptoms. Such misclassification using dietary assessment methods and diagnostic criteria would attenuate the association found in these studies, biasing the results toward the null hypothesis. The present data together with these previous studies suggest that higher coffee intake might be expected to have an inverse association with depressive symptoms.

The mechanisms behind the inverse association between coffee intake and depressive symptoms remain to be determined, but there are possible biological explanations. Coffee is a complex mixture of different chemicals that provides large amounts of caffeine, chlorogenic acid, ferulic acid, and caffeic acid.^23^ Caffeine has a strong antioxidant effect that protects against cell damage caused by lipid peroxidation in animal models,^40^ and there is evidence to suggest that oxidative stress plays an important role in the pathophysiology of anxiety.^41^ Chlorogenic acid also has anti-inflammatory and antioxidant effects,^42^^,^^43^ and owing to its central nervous system effects, it may play a part in decreasing depressive symptoms.^23^ Ferulic acid was shown to provide antioxidant protection against hydroxyl and peroxyl radical exposure,^44^ and these results are related to neurodegenerative disorders.^44^ Caffeic acid has potent antioxidant properties that are greater in antioxidant activity than many other important constituents of coffee, including chlorogenic acid and ferulic acid,^45^ and its antioxidant activities are similar to or better than that of α-tocopherol, a form of vitamin E and potent antioxidant.^46^ In this study, the association between depressive symptoms and coffee was found, but there was no association between depressive symptoms and intakes of green tea, which also includes caffeine.^35^ In addition, a non-significant but marginal inverse association was found between caffeine intake and depressive symptoms, which may partially contribute to the inverse association between coffee intake and depressive symptoms. This result implies that not only caffeine but also some other substances in coffee might associate with depressive symptoms. Because we did not assess chlorogenic acid, ferulic acid, and caffeic acid intake, we could not specifically examine the association between these substances and depressive symptoms. The inverse association between coffee intake and depressive symptoms might be caused by the combination of two or more biological active compounds, including caffeine.

A major strength of the present study includes the use of a validated dietary questionnaire and the adjustment for known and putative risk factors for depressive symptoms. Furthermore, we investigated using total caffeine intake estimated from BDHQ with foods, coffee, and green tea, since there are several sources of caffeine in foods other than coffee and green tea.^27^ However, the present study has several limitations. First, the association derived from a cross-sectional study does not indicate causality. Although we assessed the intake of coffee, green tea, and caffeine for 1 month before CES-D was conducted, reverse causation is a concern in most cross-sectional studies. There may be reverse causality in which coffee consumption decreases due to depressive symptoms. Longitudinal studies are required to confirm the present findings. Second, the validity of total caffeine intake estimated in BDHQ was not examined. However, for total caffeine intake, the Spearman correlation coefficient of the self-administered diet history questionnaire (longer version of the BDHQ) and 16-day diet records was 0.37 among 92 Japanese women (S. Sasaki, unpublished observation, 2014). A study in Taiwan reported that the correlation coefficient of caffeine using 15-day diet records was 0.30–0.56,^47^ which is similar to the findings of our study. The Spearman correlation coefficient of caffeine intake in Nurses’ Health study was high (0.76). Their study was based on 7-day records,^48^ but our study used 16-day records. The differences in correlation coefficients might be due to differences in the period of diet record. Thus, BDHQ seems to have an acceptable ability to estimate dietary caffeine intake, although the results should be interpreted with caution. Third, we assessed depressive symptoms using the CES-D questionnaire, without structured diagnostic interviews. Finally, the present findings are among elderly women whose grandchildren are students in dietetic courses at universities, colleges, and technical schools, so they might not represent the general elderly population.

## CONCLUSION

In this research, we found a significantly inverse association of coffee intake and marginal inverse association of caffeine intake with the prevalence of depressive symptoms in Japanese elderly women. Further studies are needed to determine the antidepressant effect of substances, such as chlorogenic acid, ferulic acid, and caffeic acid, or other factors related to coffee intake. The observed cross-sectional association requires confirmation in longitudinal studies.

## APPENDIX A. SUPPLEMENTARY DATA

eTable 1. Characteristics of study participants according to coffee intake: elderly Japanese women (*n* = 1,992) (Subgroup 1 = non-coffee drinkers, *n* = 491, 24.6%)

eTable 2. Adjusted odds ratio (95% CI) of depressive symptoms according to intake of coffee in elderly Japanese women (*n* = 1,992) (Subgroup 1 = non-coffee drinkers, *n* = 491, 24.6%)

## References

[1] Jeong HG, Lee JJ, Lee SB, Role of severity and gender in the association between late-life depression and all-cause mortality. Int Psychogeriatr. 2013;25:677–684. 10.1017/S1041610212002190 23256908

[2] Bromet E, Andrade LH, Hwang I, Cross-national epidemiology of DSM-IV major depressive episode. BMC Med. 2011;9(90):1–16. 10.1186/1741-7015-9-90 21791035 PMC3163615

[3] Van de Velde S, Bracke P, Levecque K Gender differences in depression in 23 European countries. Cross-national variation in the gender gap in depression. Soc Sci Med. 2010;71:305–313. 10.1016/j.socscimed.2010.03.035 20483518

[4] Niu K, Hozawa A, Kuriyama S, Green tea consumption is associated with depressive symptoms in the elderly. Am J Clin Nutr. 2009;90:1615–1622. 10.3945/ajcn.2009.28216 19828710

[5] Ruusunen A, Lehto SM, Tolmunen T, Mursu J, Kaplan GA, Voutilainen S Coffee, tea and caffeine intake and the risk of severe depression in middle-aged Finnish men: the Kuopio Ischaemic Heart Disease Risk Factor Study. Public Health Nutr. 2010;13:1215–1220. 10.1017/S1368980010000509 20359377

[6] Lucas M, Mirzaei F, Pan A, Coffee, caffeine, and risk of depression among women. Arch Intern Med. 2011;171:1571–1578. 10.1001/archinternmed.2011.393 21949167 PMC3296361

[7] Guo X, Park Y, Freedman ND, Sweetened Beverages, Coffee, and Tea and Depression Risk among Older US Adults. PLoS One. 2014;9:e94715. 10.1371/journal.pone.0094715 24743309 PMC3990543

[8] Pham NM, Nanri A, Kurotani K, Green tea and coffee consumption is inversely associated with depressive symptoms in a Japanese working population. Public Health Nutr. 2014;17:625–633. 10.1017/S1368980013000360 23453038 PMC10282314

[9] Park RJ, Moon JD Coffee and depression in Korea: the fifth Korean National Health and Nutrition Examination Survey. Eur J Clin Nutr. 2015;69:501–504. 10.1038/ejcn.2014.247 25469468

[10] Kim J, Kim J Green tea, coffee, and caffeine consumption are inversely associated with self-report lifetime depression in the Korean population. Nutrients. 2018;10:1201. 10.3390/nu10091201 30200434 PMC6163318

[11] Navarro AM, Abasheva D, Martínez-González MÁ, Coffee consumption and the risk of depression in a middle-aged cohort: the SUN project. Nutrients. 2018;10:1333. 10.3390/nu10091333 30235886 PMC6163886

[12] Grosso G, Micek A, Castellano S, Pajak A, Galvano F Coffee, tea, caffeine and risk of depression: a systematic review and dose-response meta-analysis of observational studies. Mol Nutr Food Res. 2016;60:223–234. 10.1002/mnfr.201500620 26518745

[13] Kang D, Kim Y, Je Y Non-alcoholic beverage consumption and risk of depression: epidemiological evidence from observational studies. Eur J Clin Nutr. 2018;72:1506–1516. 10.1038/s41430-018-0121-2 29500461

[14] Wang L, Shen X, Wu Y, Zhang D Coffee and caffeine consumption and depression: a meta-analysis of observational studies. Aust N Z J Psychiatry. 2016;50:228–242. 10.1177/0004867415603131 26339067

[15] Hintikka J, Tolmunen T, Honkalampi K, Daily tea drinking is associated with a low level of depressive symptoms in the Finnish general population. Eur J Epidemiol. 2005;20:359–363. 10.1007/s10654-005-0148-2 15971509

[16] Kwok MK, Leung GM, Schooling CM Habitual coffee consumption and risk of type 2 diabetes, ischemic heart disease, depression and Alzheimer’s disease: a Mendelian randomization study. Sci Rep. 2016;6:36500. 10.1038/srep36500 27845333 PMC5109212

[17] Yu ZM, Parker L, Dummer TJB Associations of coffee, diet drinks, and non-nutritive sweetener use with depression among populations in eastern Canada. Sci Rep. 2017;7:6255. 10.1038/s41598-017-06529-w 28740248 PMC5524745

[18] Hozawa A, Kuriyama S, Nakaya N, Green tea consumption is associated with lower psychological distress in a general population: the Ohsaki Cohort 2006 Study. Am J Clin Nutr. 2009;90:1390–1396. 10.3945/ajcn.2009.28214 19793850

[19] Shimbo M, Nakamura K, Jing Shi H, Green tea consumption in everyday life and mental health. Public Health Nutr. 2005;8:1300–1306. 10.1079/PHN2005752 16372926

[20] Butt MS, Sultan MT Coffee and its consumption: benefits and risks. Crit Rev Food Sci Nutr. 2011;51:363–373. 10.1080/10408390903586412 21432699

[21] McKay DL, Blumberg JB The role of tea in human health: an update. J Am Coll Nutr. 2002;21:1–13. 10.1080/07315724.2002.10719187 11838881

[22] Iso H, Date C, Wakai K, Fukui M, Tamakoshi A; JACC Study Group The relationship between green tea and total caffeine intake and risk for self-reported type 2 diabetes among Japanese adults. Ann Intern Med. 2006;144:554–562. 10.7326/0003-4819-144-8-200604180-00005 16618952

[23] Hall S, Desbrow B, Anoopkumar-Dukie S, A review of the bioactivity of coffee, caffeine and key coffee constituents on inflammatory responses linked to depression. Food Res Int. 2015;76:626–636. 10.1016/j.foodres.2015.07.027 28455046

[24] Liao S, Kao YH, Hiipakka RA Green tea: biochemical and biological basis for health benefits. Vitam Horm. 2001;62:1–94. 10.1016/S0083-6729(01)62001-6 11345896

[25] Broderick P, Benjamin AB Caffeine and psychiatric symptoms: a review. J Okla State Med Assoc. 2004;97:538–542. 15732884

[26] Smith AP Caffeine, cognitive failures and health in a non-working community sample. Hum Psychopharmacol. 2009;24:29–34. 10.1002/hup.991 19016251

[27] Yamada M, Sasaki S, Murakami K, Estimation of caffeine intake in Japanese adults using 16 d weighed diet records based on a food composition database newly developed for Japanese populations. Public Health Nutr. 2010;13:663–672. 10.1017/S1368980009992023 20082748

[28] Frary CD, Johnson RK, Wang MQ Food sources and intakes of caffeine in the diets of persons in the United States. J Am Diet Assoc. 2005;105:110–113. 10.1016/j.jada.2004.10.027 15635355

[29] Kobayashi S, Asakura K, Suga H, Sasaki S; Three-generation Study of Women on Diets and Health Study Group High protein intake is associated with low prevalence of frailty among old Japanese women: a multicenter cross-sectional study. Nutr J. 2013;12:164. 10.1186/1475-2891-12-164 24350714 PMC3878252

[30] Ministry of Health, Labour, and Welfare. *Dietary reference intakes for Japanese, 2015*. Tokyo: Ministry of Health Labour and Welfare; 2014 (in Japanese).

[31] Shima S, Shikano T, Kitamura T, Asai M Atatashii yokuutsusei jiko syouka shakudo ni tsuite [New self-rating scale for depression]. Clin Psychiatry. 1985;27:717–723 (in Japanese).

[32] Radloff L The CES-D scale: a self-report depression scale for research in the general population. Appl Psychol Meas. 1977;1:385–401. 10.1177/014662167700100306

[33] Kobayashi S, Honda S, Murakami K, Both comprehensive and brief self-administered diet history questionnaires satisfactorily rank nutrient intakes in Japanese adults. J Epidemiol. 2012;22:151–159. 10.2188/jea.JE20110075 22343326 PMC3798594

[34] Kobayashi S, Murakami K, Sasaki S, Comparison of relative validity of food group intakes estimated by comprehensive and brief-type self-administered diet history questionnaires against 16 d dietary records in Japanese adults. Public Health Nutr. 2011;14:1200–1211. 10.1017/S1368980011000504 21477414

[35] Science-and-Technology-Agency. *Standard Tables of Food Composition in Japan*. Tokyo; 2010.

[36] WC. W. *Nutritional epidemiology*. 3rd ed. New York: Oxford University; 2012.

[37] Ainsworth BE, Haskell WL, Herrmann SD, 2011 Compendium of Physical Activities: a second update of codes and MET values. Med Sci Sports Exerc. 2011;43:1575–1581. 10.1249/MSS.0b013e31821ece12 21681120

[38] Suga H, Asakura K, Kobayashi S, Nojima M, Sasaki S; Three-generation Study of Women on Diets and Health Study Group Association between habitual tryptophan intake and depressive symptoms in young and middle-aged women. J Affect Disord. 2018;231:44–50. 10.1016/j.jad.2018.01.029 29438897

[39] Fredholm BB, Bättig K, Holmén J, Nehlig A, Zvartau EE Actions of caffeine in the brain with special reference to factors that contribute to its widespread use. Pharmacol Rev. 1999;51:83–133. 10049999

[40] Devasagayam TP, Kamat JP, Mohan H, Kesavan PC Caffeine as an antioxidant: inhibition of lipid peroxidation induced by reactive oxygen species. Biochim Biophys Acta. 1996;1282:63–70. 10.1016/0005-2736(96)00040-5 8679661

[41] Rammal H, Bouayed J, Younos C, Soulimani R Evidence that oxidative stress is linked to anxiety-related behaviour in mice. Brain Behav Immun. 2008;22:1156–1159. 10.1016/j.bbi.2008.06.005 18620042

[42] dos Santos MD, Almeida MC, Lopes NP, de Souza GE Evaluation of the anti-inflammatory, analgesic and antipyretic activities of the natural polyphenol chlorogenic acid. Biol Pharm Bull. 2006;29:2236–2240. 10.1248/bpb.29.2236 17077520

[43] Natella F, Nardini M, Giannetti I, Dattilo C, Scaccini C Coffee drinking influences plasma antioxidant capacity in humans. J Agric Food Chem. 2002;50:6211–6216. 10.1021/jf025768c 12358504

[44] Kanski J, Aksenova M, Stoyanova A, Butterfield DA Ferulic acid antioxidant protection against hydroxyl and peroxyl radical oxidation in synaptosomal and neuronal cell culture systems in vitro: structure-activity studies. J Nutr Biochem. 2002;13:273–281. 10.1016/S0955-2863(01)00215-7 12015157

[45] Kikuzaki H, Hisamoto M, Hirose K, Akiyama K, Taniguchi H Antioxidant properties of ferulic acid and its related compounds. J Agric Food Chem. 2002;50:2161–2168. 10.1021/jf011348w 11902973

[46] Gülçin I Antioxidant activity of caffeic acid (3,4-dihydroxycinnamic acid). Toxicology. 2006;217:213–220. 10.1016/j.tox.2005.09.011 16243424

[47] Lee MS, Pan WH, Liu KL, Yu MS Reproducibility and validity of a Chinese food frequency questionnaire used in Taiwan. Asia Pac J Clin Nutr. 2006;15:161–169. 16672199

[48] Yuan C, Spiegelman D, Rimm EB, Validity of a dietary questionnaire assessed by comparison with multiple weighed dietary records or 24-hour recalls. Am J Epidemiol. 2017;185:570–584. 10.1093/aje/kww104 28338828 PMC5859994

