# Supplementary material for: Intake of Coffee Associated With Decreased Depressive Symptoms Among Elderly Japanese Women: A Multi-Center Cross-Sectional Study
Source: J Epidemiol. 2020 Aug 5;30(8):338–44. doi: 10.2188/jea.JE20190010 (PMC7348072; doi:10.2188/jea.JE20190010)
Supplement: Supplementary file 1 [file je-30-338-s001.pdf]

eTable 1. Characteristics of study participants according to coffee intake: elderly Japanese women (n=1,992) (Subgroup 1= non-coffee drinkers, n=491, 24.6%)

	Coffee intake				
	Subgroup 1 (n=491)	Subgroup 2 (n=500)	Subgroup 3 (n=501)	Subgroup 4 (n=500)	Trend *P* [a]
CES-D score	11.9 (7.5) [b]	10.7 (6.3)	11.0 (6.3)	10.2 (6.5)	0.0004
Median intake, g/1,000 kcal	0 (0–0) [c]	19 (3–57)	80 (58–106)	193 (107–619)	
Age, years	76.4 (5.3)	74.8 (4.8)	74.4 (4.6)	73.0 (4.5)	<0.0001
BMI, kg/m^2	22.4 (3.1)	23.1 (3.1)	22.9 (3.2)	22.6 (3.1)	0.38
Residential block, %					
Hokkaido and Tohoku	10.6	7.8	11.2	6.8	0.008
Kanto	30.6	31.6	22.6	16.0	
Hokuriku and Tokai	21.6	22.8	24.2	27.0	
Kinki	7.8	11.2	14.8	16.8	
Chugoku and Shikoku	10.0	10.4	17.8	25.4	
Kyushu	19.6	16.2	9.6	8.0	
Size of residential area, %					
City with a population ≥1 million	10.0	14.0	15.2	12.8	0.24
City with a population <1 million	78.6	75.4	73.3	76.8	
Town and village	11.4	10.6	11.6	10.4	
Married, %	60.3	58.4	61.5	63.2	0.23
Living status (alone), %	18.5	14.4	17.4	15.0	0.31
Physical activity, METs/day	37.7 (6.5)	39.1 (6.3)	39.1 (6.2)	40.0 (6.4)	<0.0001
Current smoking, %	1.8	2.0	2.2	3.8	0.05
Current alcohol intake, %	10.8	19.0	23.6	24.0	<0.0001
Education, %					
Junior high school	49.1	49.0	44.5	41.6	0.005
High school	44.0	41.4	45.3	48.0	
Junior college	5.7	9.0	8.8	9.2	
University or higher	1.2	0.6	1.4	1.2	
Caffeine intake, mg/1,000 kcal [d]	142.1 (80.6)	152.0 (76.6)	170.7 (69.3)	260.5 (95.5)	<0.0001
EPA+DHA intake, mg/1,000 kcal	669.0 (390.3)	658.0 (330.9)	644.5 (353.7)	580.3 (303.7)	<0.0001
Folate intake, µg/1,000 kcal	245.0 (82.3)	243.3 (70.6)	237.4 (68.4)	232.3 (71.9)	0.003
Dietary supplement use, %	25.5	30.4	30.5	34.0	0.005

BMI, body mass index; CES-D, Center for Epidemiologic Studies Depression Scale; DHA, docosahexaenoic acid; EPA, eicosapentaenoic acid; METs, metabolic equivalent hours.
Participants with depressive symptoms were defined as a CES-D score ≥16.
[a] Trend *P* values were based on linear regression analysis for continuous variables with ordinal numbers 0–3 assigned to green tea and coffee intake categories, or Mantel-Haenszel chi-square test for categorical variables.
[b] Values for continuous variables are in mean ± standard deviation (all such values).
[c] Range.
[d] Calculated from green tea, black tea and Chinese tea, coffee, and cola.

eTable 2. Adjusted odds ratio (95% CI) of depressive symptoms according to intake of coffee in elderly Japanese women (n=1,992)
(Subgroup 1 = non-coffee drinkers, n=491, 24.6%)

	Coffee intake				
	Subgroup 1 (n=491)	Subgroup 2 (n=500)	Subgroup 3 (n=501)	Subgroup 4 (n=500)	Trend *P* [a]
Median intake, g/1,000 kcal	0 (0–0)	19 (3–57)	80 (58–106)	193 (107–619)	
Depressive symptoms, %	28.7	20.2	21.0	18.4	
Crude OR (95% CI)	1.00 (Reference)	0.63 (0.47-0.84)	0.66 (0.49-0.88)	0.56 (0.42-0.76)	0.0003
Age adjusted OR (95% CI)	1.00 (Reference)	0.67 (0.50-0.90)	0.71 (0.53-0.96)	0.64 (0.47-0.87)	0.008
Model 1 [b] OR (95% CI)	1.00 (Reference)	0.68 (0.50-0.92)	0.73 (0.54-0.99)	0.66 (0.48-0.91)	0.02
Model 2 [c] OR (95% CI)	1.00 (Reference)	0.70 (0.52-0.95)	0.75 (0.55-1.02)	0.65 (0.47-0.89)	0.01
Model 3 [d] OR (95% CI)	1.00 (Reference)	0.70 (0.52-0.95)	0.73 (0.54-1.00)	0.64 (0.46-0.88)	0.01

BMI, body mass index; CES-D, Center for Epidemiologic Studies Depression Scale; CI, confidence interval; DHA, docosahexaenoic acid; EPA, eicosapentaenoic acid; METs, metabolic equivalent hours; OR, odds ratio.
Participants with depressive symptoms were defined as a CES-D score ≥16.
Green tea, coffee intake, EPA + DHA intake and folate intake were energy-adjusted according to the density method.
[a]Trend *P* values were based on linear regression analysis for continuous variables with ordinal numbers 0–3 assigned to green tea or coffee intake categories.
[b]Model 1: adjusted for age (y, continuous) and residential block (Hokkaido and Tohoku, Kanto, Hokuriku and Tokai, Kinki, Chugoku and Shikoku, and Kyushu), living status (alone or not alone), current smoking (yes or no), alcohol drinking (yes or no), marital status (married or unmarried), physical activity level (total metabolic equivalents hours/day: METs, continuous), size of residential area (city with a population ≥1 million, city with a population <1 million, and town and village), BMI (kg/m^2, continuous) and education (junior high school, high school junior college, and university and higher).
[c]Model 2: adjusted for variables in model 1 with EPA + DHA intake (mg/1,000 kcal, continuous), folate intake (μg/1,000 kcal, continuous), dietary supplement use (yes or no).
[d]Model 3: adjusted for variables in model 2 with mutually adjusted for green tea intake (g/1,000 kcal) or coffee intake (g/1,000 kcal).
